# Perihippocampal Meningeal Carcinomatosis Following Hippocampal Avoidance Prophylactic Cranial Irradiation in Small Cell Lung Cancer: A Case Report

**DOI:** 10.7759/cureus.46499

**Published:** 2023-10-04

**Authors:** Masateru Fujiwara, Hirohito Tada

**Affiliations:** 1 Radiation Oncology, Osaka University Graduate School of Medicine, Suita, JPN; 2 Radiation Oncology, Suita Tokushukai Hospital, Suita, JPN; 3 Thoracic Surgery, Suita Tokushukai Hospital, Suita, JPN

**Keywords:** side effects of radiotherapy, radiotherapy (rt), small cell lung cancer (sclc), intensity modulated radiation therapy (imrt), radiation oncology neurosurgery, neurocognitive side effects, meningeal carcinomatosis, radiation and clinical oncology, whole-brain radiotherapy, small-cell lung carcinoma

## Abstract

Prophylactic cranial irradiation (PCI) for limited disease small cell lung cancer is the standard of care for curative treatment of this disease. However, neurocognitive dysfunction is one of the late adverse events of PCI and is often problematic. Recently, hippocampal avoidance prophylactic cranial irradiation (HA-PCI) is sometimes performed to prevent neurocognitive dysfunction after PCI. In HA-PCI, the question is whether or not metastases appear around the hippocampus that were not irradiated. We have experienced a case of perihippocampal meningeal carcinomatosis after HA-PCI. We also draw attention to the potential risks of performing HA-PCI based on this experience.

## Introduction

Throughout the entire world, prophylactic cranial irradiation (PCI) is the standard of care to improve both overall survival and disease-free survival among patients with limited disease small cell lung cancer (LD-SCLC) in complete remission after concurrent thoracic chemoradiotherapy [1, 2]. As a matter of caution, one of the late adverse events of whole-brain irradiation, including PCI, is radiation-induced neurocognitive function (NCF) deficit, which is thought to be related to hippocampal injury [3, 4, 5]. Recently, evidence of hippocampal avoidance PCI (HA-PCI) using intensity-modulated radiation therapy (IMRT) or volumetric-modulated arc therapy (VMAT) has been reported with the aim of better preserving NCF [6, 7]. For example, the GICOR-GOECP-SEOR study demonstrated its effectiveness in a randomized controlled trial [7]. However, sparing the hippocampus poses the theoretical risk of intracranial metastatic progression in the perihippocampal region. This report describes a case of perihippocampal meningeal carcinomatosis after HA-PCI, and we also draw attention to the potential risks of performing HA-PCI based on this experience. To the best of our knowledge, this is the first report of perihippocampal meningeal carcinomatosis in patients who underwent HA-PCI to treat LD-SCLC.

## Case presentation

**Figure 1:**
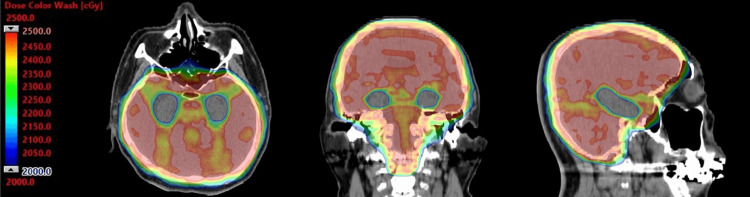
Dose distribution map of hippocampal avoidance prophylactic cranial irradiation. The images display the dose distribution map of hippocampal avoidance prophylactic cranial irradiation (HA-PCI). The range of color wash doses is 20Gy to 25Gy. HA-PCI was performed using volumetric-modulated arc therapy (VMAT) technology with 3 arcs. The VMAT plan was developed using the Eclipse treatment planning system (version 11.0, Varian Medical Systems) with the Acuros XB algorithm for heterogeneity correction. The treatment plans were generated and delivered utilizing 6-MV photon beams from a TrueBeam™ STx linear accelerator (Varian Medical Systems, Palo Alto, CA, USA), equipped with 2.5 mm multileaf collimators.

A 55-year-old male patient diagnosed with LD-SCLC underwent initial treatment involving concurrent chemoradiotherapy with cisplatin (CDDP) and etoposide (VP16). He received twice-daily thoracic radiation therapy, totaling 45 Gy in 30 fractions, via accelerated hyper-fractionated radiation therapy (AHF-RT). Three months later, the response was assessed, and the chest lesion was deemed to be in complete remission (CR). At that point, a contrast-enhanced brain MRI was performed; no brain metastases were noted, so PCI was elected to be performed. Based on recent evidence [7], HA-PCI using VMAT was irradiated at 25 Gy in 10 fractions. (Figure 1, Table 1). However, three months later, bilateral adrenal gland metastases were discovered, and the disease was classified as progressive disease (PD). Consequently, systemic chemotherapy was initiated. One year after HA-PCI, the patient's cognitive function, evaluated using the Mini-Mental State Examination, yielded a score of 29 points, revealing no abnormalities in NCF. At that time, the disease status was determined to be stable disease (SD), and the patient's performance status was deemed good.

**Table 1:** Dose-volume parameters of hippocampal avoidance prophylactic cranial irradiation. ^a^PTV: Planning target volume.

Structure	Evaluation index	Dose (cGy)
PTV ^a^	D50_%_	2500
PTV ^a^	D_MAX_	2647
Hippocampus	D_MAX_	1534
Hippocampus	D_0.1cc_	1215
Hippocampus	D_mean_	987
Hippocampus	D100_%_	716
Chiasm	D_MAX_	2529
Left optic nerve	D_MAX_	2500
Right optic nerve	D_MAX_	2476
Left lens	D_0.1cc_	478
Right lens	D_0.1cc_	457
Brain stem	D_0.1cc_	2515
Left cochlea	D_MAX_	2511
Right cochlea	D_MAX_	2493

**Figure 2:**
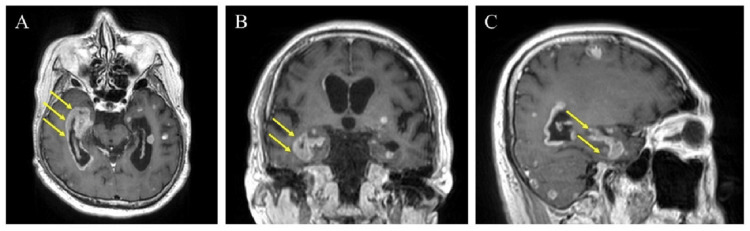
MRI images of meningeal carcinomatosis in the perihippocampus following hippocampal avoidance prophylactic cranial irradiation. The images show axial (A), coronal (B), and sagittal (C) contrast-enhanced T1-weighted images. Meningeal carcinomatosis is visible as solid enhancing masses in the perihippocampus (arrows in A-C).

However, a contrast-enhanced brain MRI performed two months later revealed multiple brain metastases and lesions suspicious of meningeal dissemination in the lateral ventricle around the right hippocampus. Gamma knife therapy (GK) irradiated at 20 Gy with a marginal dose prescription was performed on each of the nine brain metastases around the motor cortex to prevent motor paralysis. One month after GK, the patient was rushed to the hospital due to lower limb weakness, and a contrast-enhanced brain MRI was performed on the same day. The MRI images revealed worsening multiple brain metastases and meningeal carcinomatosis, primarily in the lateral ventricles around the hippocampus (Figure 2). Betamethasone administration and whole brain re-irradiation at 25 Gy in 10 fractions were urgently performed. However, one week later, his level of consciousness suddenly decreased to a score of 3 on the Glasgow Coma Scale (GCS). The cause of unconsciousness was thought to be neoplastic meningitis. The patient died a few days later.

**Figure 3:**
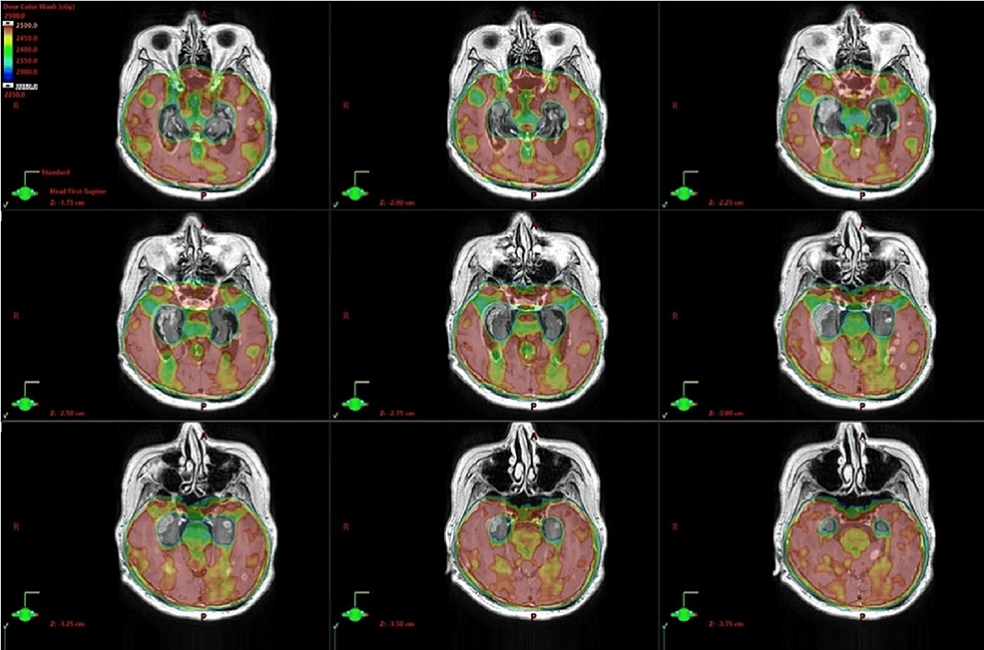
Registration image: MRI of meningeal carcinomatosis and dose distribution map for hippocampal avoidance prophylactic cranial irradiation. The images display multi-slice axial registration, which fuses the magnetic resonance images of meningeal carcinomatosis with the dose distribution map of hippocampal avoidance prophylactic cranial irradiation. The color wash dose range is 22.5Gy to 25Gy. These registration images, created using the Eclipse treatment planning system (version 16.0, Varian Medical Systems), clearly show that the area avoiding the hippocampus overlaps with the main lesion of meningeal carcinomatosis.

Based on the fusion images of the contrast-enhanced MRI image when meningeal carcinomatosis was diagnosed and the dose distribution map of HA-PCI, it was thought that the disease had developed from the area around the hippocampus that was avoided by HA-PCI (Figure 3).

## Discussion

Several literatures have reported that avoiding the hippocampus in PCI preserves NCF [6-8]. In the present case, NCF was certainly preserved one year after PCI, owing to the treatment with HA-PCI, and HA-PCI might have been effective in preserving NCF, as reported. In a previous major randomized controlled trial (RCT), Faivre-Finn C et al. reported a median survival of 30 months and a two-year survival rate of 56% after concurrent chemoradiotherapy with AHF-RT in combination with CDDP+VP-16 for LD-SCLC [9]. However, this case did not achieve the reported median survival for LD-SCLC. While the patient's NCF was preserved by avoiding the hippocampus, the prognosis was worsened by the peri-hippocampal meningeal carcinomatosis. Generally, the risk of developing meningeal carcinomatosis in solid tumors is 5-10%. Advances in chemotherapy have prolonged the overall survival of patients with advanced cancer, and consequently, the number of people developing meningeal carcinomatosis is increasing. Unfortunately, the prognosis for those patients who progress to neoplastic meningitis is poor, with survival ranging from 8 to 16 weeks [10].

Kundapur V et al. concluded that the risk of developing metastases in the perihippocampal region after hippocampal avoidance cranial irradiation in patients affected by SCLC is 5%, and the overall incidence of metastases in the hippocampal avoidance region before or after HA-PCI in SCLC patients was low [11]. Certainly, HA-PCI is effective in terms of preserving NCF. However, when meningeal carcinomatosis unfortunately develops around the hippocampus after HA-PCI, as in the present case, there are few effective salvage therapies. In our opinion, it is still controversial to determine the previously described 5% as low risk. It is unclear how HA-PCI will be positioned in future LD-SCLC treatment methods. However, at this time, we would like to emphasize the need for careful CNS follow-up after HA-PCI because brain metastases could occur even after PCI in SCLC, and meningeal carcinomatosis could develop in the non-irradiated hippocampal region after HA-PCI, as in this case.

## Conclusions

We have reported a case of meningeal carcinomatosis in the perihippocampal regions after HA-PCI. Although HA-PCI might effectively preserve NCF, the possibility that perihippocampal recurrence poses a fatal risk to patients should also be considered. We emphasize the need for careful CNS follow-up after HA-PCI.
